# Cocrystallization of Ezetimibe with Organic Acids: Stoichiometric Optimization for Improved Solubility and Bioavailability

**DOI:** 10.3390/pharmaceutics17111399

**Published:** 2025-10-29

**Authors:** Ravi Maharjan, Ha Eun Park, Ki Hyun Kim, Mansingh Chaudhary, Ki-Taek Kim, Minji Kim, Hea-Young Cho, Seong Hoon Jeong

**Affiliations:** 1 College of Pharmacy & Yonsei Institute of Pharmaceutical Sciences, Yonsei University, Incheon 21983, Republic of Korea; ravimaharjan@yonsei.ac.kr; 2 College of Pharmacy, Dongguk University, Goyang 10326, Republic of Korea; haeun9065@dgu.ac.kr; 3 College of Pharmacy and Natural Medicine Research Institute, Mokpo National University, Muan 58554, Republic of Korea; kihyunkim@mnu.ac.kr (K.H.K.); ktkim0628@mnu.ac.kr (K.-T.K.); 4 Department of Biomedicine, Health & Life Convergence Sciences, BK21 Four, Biomedical and Healthcare Research Institute, Mokpo National University, Muan 58554, Republic of Korea; mansingh9607@gmail.com; 5 College of Pharmacy, CHA University, Seongnam 13488, Republic of Korea; kmj6296@gmail.com (M.K.); hycho@cha.ac.kr (H.-Y.C.)

**Keywords:** pharmaceutical cocrystallization, organic acids, solubility enhancement, solvent evaporation, solvent/anti-solvent method

## Abstract

**Background/Objectives:** Pharmaceutical cocrystallization offers a promising strategy to enhance drug properties while preserving molecular integrity. Ezetimibe, a BCS Class II hypolipidemic agent, faces therapeutic limitations due to poor aqueous solubility. This study aimed to systematically evaluate cocrystallization of ezetimibe with organic acid (benzoic, tartaric, or succinic acid) at varying stoichiometric ratios (1:0.5–1:2) to optimize physicochemical properties and oral bioavailability. **Methods:** Cocrystals were prepared via solvent evaporation (SEV) and solvent/anti-solvent (SAS) methods. Structural characterization included Fourier-transform infrared spectroscopy (FTIR), differential scanning calorimetry (DSC), and powder/single-crystal X-ray diffraction (PXRD/SCXRD). Physicochemical performance was assessed through saturation solubility, in vitro dissolution, and in vivo pharmacokinetics in male Sprague Dawley rats (*n* = 4/group). **Results:** Benzoic acid cocrystals (1:2 ratio, SEV) showed O−H⋯N hydrogen bonding (FTIR band shifts: 2928 → 3264 cm^−1^) and novel crystalline phases (12.4°, 16.7°, and 24.9°). SCXRD confirmed monoclinic P2_1_/n symmetry (a = 5.42 Å, b = 5.05 Å) for benzoic acid cocrystals. Ezetimibe/benzoic acid cocrystals (1:2) achieved 64-fold solubility enhancement and 2× faster dissolution vs. pure ezetimibe. Pharmacokinetics revealed 3× higher Cmax (18.38 ng/mL) and 4× greater AUC (40.36 h·ng/mL) for optimized cocrystals. Tartaric and succinic acid cocrystals showed moderate improvements, with melting points intermediate between parent compounds. **Conclusions:** Both stoichiometry and preparation method strongly determined cocrystal performance. Benzoic acid at a 1:2 ratio via SEV demonstrated superior solubility, dissolution, and bioavailability, addressing ezetimibe’s formulation challenges. These findings underscore the potential of rational cocrystal design to overcome solubility barriers in oral dosage development, particularly for hydrophobic therapeutics.

## 1. Introduction

In conventional tablet or capsule formulations, active pharmaceutical ingredients (APIs) are typically incorporated as crystalline solids [1]. In the preliminary stages of oral dosage formulation development, the crystalline form of API should be determined [2]. The bioavailability of hydrophobic APIs, classified as BCS Class II or IV, is significantly impeded by their low aqueous solubility and dissolution rate [3]. Pharmaceutical cocrystallization can produce various solid forms of an API, facilitating the optimization of key drug properties [4,5]. This well-established method enhances the biopharmaceutical properties of APIs without the need for covalent modifications [6]. Cocrystals are categorized as molecular or ionic, each offering unique compositional and physicochemical advantages [7,8,9]. The systematic study of pharmaceutical cocrystals gained momentum in the 2000s and has now reached an important phase, demonstrating their potential to enhance the physicochemical properties of APIs and even improving their therapeutic effects [10]. Recent nanoparticle advances underscore the need for integrated in vivo validation, which our study addresses [11,12]. To ensure the synthesized sample is stable and its therapeutic efficacy is consistent, we proposed to conduct further studies such as solubility study, in vitro release profile, phase diagram, crystal identification, microscopic imaging, and pharmacokinetic evaluation on rats. In this study, we investigated the cocrystallization of ezetimibe, a hypolipidemic agent used to lower cholesterol levels, as a model drug. Ezetimibe inhibits the absorption of cholesterol in the small intestine, thereby reducing the amount of cholesterol delivered to the liver. It is used for the treatment and prevention of atherosclerosis [8]. Ezetimibe’s hydrophobicity limits aqueous solubility and bioavailability [13]. Incorporating hydrophilic coformers into the cocrystals of hydrophobic APIs has been shown to enhance drug solubility and in vitro release profiles [14,15].

The objective of the study is to comprehensively explore the suitable coformer for the specific drug compound, optimize preparation method, and improve the pharmaceutical attributes. Firstly, we aim to examine the molecular determinants and design principles of coformer pairs that facilitate the formation of cocrystals. This primary objective is challenging because API-improving cocrystals are rare, isolated systems, and predictive design rules have yet to be established [16]. This study aimed to (i) identify suitable coformers for ezetimibe, (ii) optimize preparation conditions for stable cocrystals, and (iii) evaluate their physicochemical/pharmacokinetic performance [17].

## 2. Materials and Methods

### 2.1. Materials

Ezetimibe (CAS no. 163222-33-1, melting point 163 °C) was purchased from Zhejiang Peptides (Zhejiang Peptides, Shengzhou, China). Benzoic acid, tartaric acid, succinic acid, sodium lauryl sulfate (SLS), polyethylene glycol (PEG) 8000, cetyltrimethylammonium bromide (CTAB), and ethyl acetate were procured from Daejung Chemicals & Metals (Siheung, Republic of Korea). Transcutol^®^ HP was purchased from Gattefosse (Saint–Priest, France). Tween 80 was purchased from Sigma–Aldrich (St. Louis, MO, USA). Methanol (MeOH—HPLC grade) was purchased from J.T. Baker^®^ (Phillipsburg, NJ, USA).

### 2.2. Preparation of Cocrystals with Solvent Evaporation (SEV) Method

Ezetimibe and each coformer (benzoic acid, tartaric acid, and succinic acid) were dissolved in ethyl acetate at weight ratios of 1:0.5, 1:1, and 1:2 (Figure S1). These solutions were heated to 70 °C on a hot plate while stirring until a clear solution was obtained. The mixtures were dried in an oven at 30 °C for 7 days.

### 2.3. Preparation of Cocrystals with Solvent/Anti—Solvent (SAS) Method

Ezetimibe and each coformer were separately dissolved in MeOH (solvent) at molar ratios of 1:0.5, 1:1, and 1:2 (Figure S1). These solutions were gradually added dropwise to water (anti-solvent) at 25 °C while stirring at 300 rpm [18]. The mixtures were then dried in an oven set at 30 °C for 7 days. The cocrystals formed using the SEV method were denoted as “M1,” while those formed using the SAS method were denoted as “M2”.

### 2.4. Preparation of Single Crystal

Ezetimibe and each coformer were mixed at a molar ratio of 1:1 and dissolved in 20 mL MeOH. The sample was prepared in a glass Petri dish and stirred until a clear solution was obtained. It was then slowly dried at 25 °C without stirring until the solvent completely evaporated. The obtained single cocrystal samples were denoted as EBA (ezetimibe/benzoic acid), ETA (ezetimibe/tartaric acid), and ESA (ezetimibe/succinic acid). One hundred mg sample was prepared for the analysis. The prepared cocrystal was finally stored in a desiccator. Cocrystal stoichiometry was quantified by high-performance liquid chromatography (HPLC) method, as described in a prior article [19].

### 2.5. Morphology and Spectroscopy

The morphology of the cocrystals was analyzed under microscope (Olympus Corporation, Tokyo, Japan) using a software provided from the manufacturer. Additionally, the samples were further examined via scanning electronic microscope (SEM) (CLARA LMH, Tescan, Brno, Czech Republic). The samples were subjected to Fourier-Transform Infrared Spectroscopy (FTIR, Thermo Fisher Scientific, Waltham, MA, USA) over a wavenumber region of 4000–500 cm^−1^, with 32 scan rates at a resolution of 4 cm^−1^. The data collected was analyzed using the OMNIC paradigm software.

### 2.6. Differential Scanning Calorimetry (DSC)

The samples were subjected to DSC using DSC Q2000 (TA Instruments, New Castle, DE, USA). The samples (2–5 mg) were placed in non-hermetic aluminum pans and heated from 25 to 200 °C at a scan rate of 5 °C/min. The thermal data were analyzed using the software provided with the Instruments [20].

### 2.7. Powder X-Ray Diffraction (PXRD) and Single-Crystal X-Ray Diffraction (SCXRD)

The PXRD patterns were measured using D2 phaser benchtop X-ray diffractometer (Ultima IV, Rigaku, Tokyo, Japan) equipped with a Ni–filtered Cu–Kα laser (λ = 1.54056 Å) and a scintillation center detector. The powder samples were placed in a quartz holder and scanned over a range of 4–40° at a scanning rate of 6°/min. SCXRD patterns were measured at −173.15 °C using Bruker D8 Venture (Billerica, MA, USA). Single crystal X-ray diffraction data were collected on a Bruker−AXS SMART APEX CCD diffractometer with monochromatized Mo Kα radiation (λ = 0.71073 Å) connected to a low-temperature device. Data was collected at 100 K. Lattice parameters were determined from least-squares analysis, and reflection data were integrated using the program SAINT. Lorentz and polarization corrections were applied for diffracted reflections. The structures were solved by direct methods and refined by full matrix least-squares.

### 2.8. Bioanalytical Method Development

The stock standard solution was prepared by dissolving ezetimibe at 0.5 mg/mL in 40% MeOH. The absorbance of the standards was measured at 233 nm using UV-VIS spectrophotometer (Optizen pop, Mecasys^®^, Seoul, Republic of Korea). The bioanalysis was performed using validated LC-MS/MS methods with MRM detection (details in Supplementary Materials). The method was developed using an Agilent 1290 Infinity II BioLC system coupled with an Agilent 6495D Triple-Quadrupole mass spectrometer, employing electrospray ionization (ESI). Chromatographic separation was achieved on a Kintex C18 column (2.6 μm, 100 × 3 mm) maintained at 30 °C, using an isocratic mobile phase of water-acetonitrile (20:80 *v*/*v*) at 0.25 mL/min flow rate. The run time was optimized to 5 min with a 2 μL injection volume. Mass spectrometric detection utilized multiple reaction monitoring (MRM) in negative polarity mode for ezetimibe ([M − H]^−^ *m*/*z* 408.1 → 271.1) and positive mode for the internal standard itraconazole ([M + H]^+^ *m*/*z* 705.2 → 391.6). Detailed description is included in the Supplementary Materials. The method demonstrated linearity (R^2^ = 0.995) across 1–100 ng/mL. Quality control samples at four concentrations (1, 3, 40, and 80 ng/mL) showed acceptable accuracy (88.47–103.08%) and precision (CV ≤ 9.8%).

### 2.9. Solubility and Dissolution

The solvents considered for the solubility study were 0.5% *w*/*v* SLS, 0.5% *w*/*v* PEG 8000, 0.5% *w*/*v* Transcutol^®^ HP, and 0.1% *w*/*v* CTAB. The solubility was assessed with ezetimibe and 1:0.5 ratio samples. The samples were mixed in a 5 mL tube with 2 mL of the solvent at a concentration of 1 mg/mL using a multi-mixer (SLRM-3, Seoulin Biosciences, Pangyo, Republic of Korea) for 48 h at 25 °C. The mixtures were then centrifuged at 13,000 rpm for 10 min (Eppendorf 5425R, Hamburg, Germany) and manually filtered using a 0.45 μm polytetrafluoroethylene (PTFE-H) syringe filter. The absorbance of the filtered samples at 233 nm was measured using a UV-VIS spectrophotometer. Similarly, dissolution was assessed using the USP dissolution apparatus II (paddle) (Varian 705 DS, Cary, NC, USA) at 50 rpm in 500 mL of 0.45% *w*/*v* SLS in 0.05 M acetate buffer (pH 4.5) at 37 ± 0.5 °C. The cocrystal samples (equivalent to 10 mg ezetimibe) were weighed and added into the dissolution vessel (n = 3). Aliquots were collected at predetermined time points: 5 min, 10 min, 15 min, 30 min, 45 min, 60 min, 90 min, and 120 min. The displaced volume was replenished with fresh buffer after each sampling. The collected samples were filtered through a 0.45 µm RC syringe filter and subjected to UV-VIS spectrophotometer.

### 2.10. In Vivo Pharmacokinetics

In vivo pharmacokinetic profile of ezetimibe cocrystal formulations was assessed in male Sprague Dawley rats (n = 4 per group, 7–8-week-old, mean weight 271.1 g). The SD rats were obtained from G-Bio (Gwangju, Republic of Korea). They were maintained under a 12 h light/dark cycle and provided with free access to food and water. The animal experimental protocol was approved by the Institutional Animal Care and Use Committee (IACUC) of Mokpo National University (Mokpo, Republic of Korea; approval no. MNU-IACUC-2025-015).

The rats were fasted overnight before oral administration, and four formulation groups received single oral doses through gavage: Group 1 (CRYS101) at 10 mg/kg and Groups 2–4 (CRYS102-104) at 30 mg/kg. Blood samples (200 μL) were collected from the femoral artery at predetermined time points after the administration. The collected samples were centrifuged at 16,000× *g* for 5 min at 4 °C, and the plasma was stored at −70 °C until analysis. The plasma samples analyzed using a validated UPLC-MS/MS method with 1 ng/mL lower quantification limit. The bioanalytical method demonstrated acceptable validation parameters with 88–103% accuracy and ≤9.8% precision across the calibration range (1–100 ng/mL), utilizing protein precipitation with acetonitrile (3.75:1 solvent: plasma ratio) and isocratic chromatographic separation (ACN:H_2_O = 80:20).

### 2.11. Statistical Analysis

The data are expressed as mean ± standard deviation (SD). Statistical analysis was performed using MS Excel (Microsoft Corporation, Malvern, PA, USA), with comparisons of means conducted using Student’s *t*-test. A *p*-value < 0.05 was considered statistically significant.

## 3. Results and Discussion

### 3.1. Thermodynamic Properties

Thermal events in the DSC provided key preliminary information on the presence of a new solid phase. The thermograms of ezetimibe, benzoic acid, tartaric acid, succinic acid, and the corresponding cocrystals are shown in Figure S3. Ezetimibe, benzoic acid, tartaric acid, and succinic acid exhibited single melting endotherms (T_m_) (see Table 1), which were consistent with the previously reported values [21,22,23,24]. EBA cocrystals showed large ΔT_m_ shifts (−50 °C vs. API), consistent with amorphous characteristics, while ETA showed moderate compatibility (−2 °C to −8 °C shifts), and ESA displayed poor compatibility. The temperature change (ΔT_m_, API vs. benzoic acid) is 39.17 °C. Although the enthalpy change in physical mixture was in a range of 110–111 °C, similar to that of SEV/SAS method, another second T_m_ near API’s original T_m_ (162 °C) indicate phase separation. Similarly, samples that of SEV/SAS methods have moderate enthalpy (50–120 J/g), while physical mixtures have lower values (8–56 J/g). Physical blends have weak interactions. In case of samples with tartaric acid, the temperature change (ΔT_m_, API vs. tartaric acid) is −10.31 °C, indicates better compatibility. When both API and coformer were incorporated into formulations using SEV/SAS method, first T_m_ was 154–160 °C (see Table 1) and second ΔT_m_ near coformer’s T_m_ (166–168 °C), which suggests possible cocrystal formation. The physical mixture in a range of 170–171 °C, indicates coformer stability (−2 °C vs. coformer). Similarly, samples that of SAS method showed increasing enthalpy in second melt (up to 144 J/g), and physical mixtures also exhibited high second-melt enthalpy (143–189 J/g), indicating stronger interactions in cocrystal phases. Similarly, the temperature change (ΔT_m_, API vs. succinic acid) is −26.55 °C, which implies poor compatibility. When API and coformer were mixed using SEV/SAS method, ΔT_m_ change was in the range of 183–188 °C (−5 °C vs. coformer), which indicates partial stabilization near coformer’s T_m_. The physical mixture indicates partial stabilization near coformer’s T_m_. Second T_m_ at 183–186 °C implies no significant improvement over SEV/SAS methods. Enthalpy increases drastically in second melt (up to 231 J/g for SEV), suggesting coformer’s thermal behavior dominance in later stages.

Most of the cocrystals exhibited two endothermic peaks, indicating the formation of a new phase [25]. Changes in enthalpy between the two peaks were also observed depending on the ratio of API/coformer. The peak thermograms of the physical mixtures were like those of the corresponding cocrystals. This was attributed to the minor interactions between the coformer and the API, leading to weaker intensity peaks comparable to those of the corresponding cocrystals [26]. In a previous screening of 50 distinct cocrystal samples, 26 samples (51%) showed melting points between those of the API and the coformer, 19 (39%) showed lower melting points, 3 (6%) showed higher melting points, and 2 (4%) showed melting point identical to either the API or the coformer [27].

### 3.2. Physicochemical Interactions

**Figure 1:**
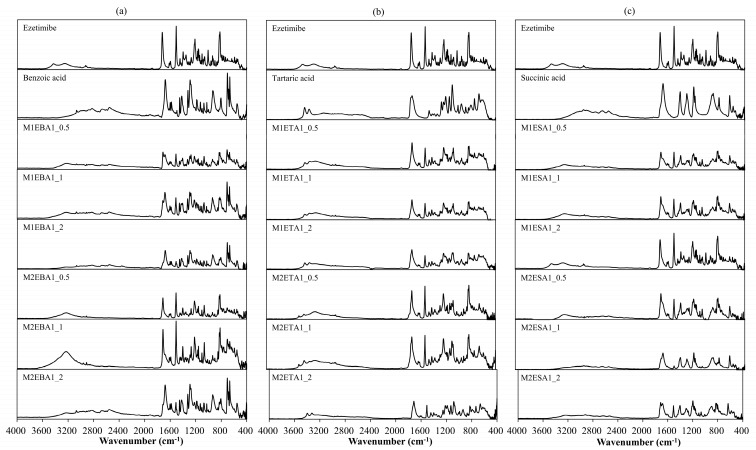
FTIR spectra of (**a**) ezetimibe/benzoic acid, (**b**) ezetimibe/tartaric acid, and (**c**) ezetimibe/succinic acid cocrystals. The spectra of the parent compound obtained using solvent evaporation and anti-solvent precipitation methods at 1:0.5, 1:1, and 1:2 ratios.

FTIR analysis (Figure 1) confirmed the interaction between ezetimibe and the employed coformers, showing main peaks and the characteristic shifts for ezetimibe, EBA, ETA, and ESA (Table 2). The formation of cocrystals induced several significant changes. The FTIR spectrum of ezetimibe showed the O–H stretching band at 3264.09 cm^−1^, which may have overlapped with the N–H stretching band. The FTIR spectra of the cocrystals showed broader peaks that shifted to lower wavelengths. The C–H stretching band at 2928.09 cm^−1^ was corresponded to API. However, the C–H band was not observed in the spectra of M1EBA1_1, M1EBA1_2, M2EBA1_2, and ETA cocrystals. The C=O stretching band corresponding to carboxylic acids was observed at 1726.06 cm^−1^, appearing at similar or lower wavelengths in the spectra of the cocrystals. The C=C stretching band corresponding to benzene was observed at 1507.08 cm^−1^. In the spectra of the cocrystals, this band appeared at a similar wavelength but was more pronounced and narrower. The C–F stretching band, indicating fluorination, was observed at 1212.71 cm^−1^. The intensity of this peak was reduced in the cocrystals. Characteristic shifts in O–H (3264 → 3224 cm^−1^), C=O (1726 → 1712 cm^−1^), and C–F (1212 → 1219 cm^−1^) bands confirmed hydrogen bonding and altered molecular interactions (Table 2).

### 3.3. Crystallinity

**Figure 2:**
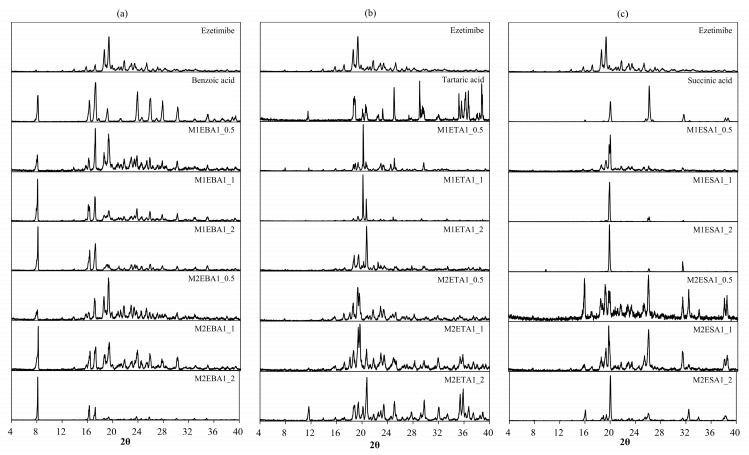
PXRD patterns of the (**a**) ezetimibe/benzoic acid, (**b**) ezetimibe/tartaric acid, and (**c**) ezetimibe/succinic acid cocrystals. The patterns of the parent compound were obtained using solvent evaporation and anti-solvent precipitation methods at 1:0.5, 1:1, and 1:2 ratios.

**Figure 3:**
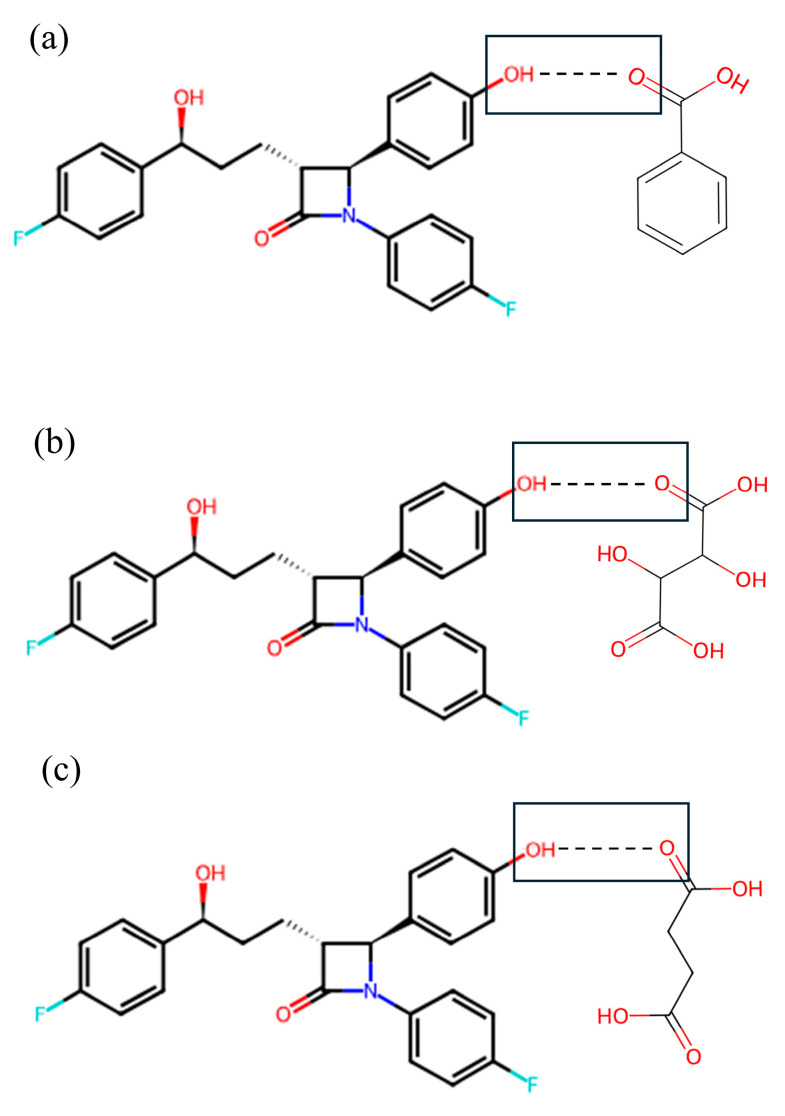
Expected motif (OH–O) hetero-synthon formation between (**a**) -OH group of ezetimibe and -C=O group of benzoic acid, (**b**) -OH group of ezetimibe and -C=O group of tartaric acid, and (**c**) -OH group of ezetimibe and -C=O group of succinic acid. The structure of single crystals comprising ezetimibe, benzoic acid, tartaric acid, succinic acid, and methanol was determined by SCXRD analysis (Table 3). The corresponding crystallographic parameters are provided in Table 3. The analysis indicated that EBA crystallizes in a monoclinic system with space group P2_1_/n and lattice parameters a = 5.42 Å, b = 5.05 Å, c = 21.61 Å, and angles α = 90°, β = 95.95°, γ = 90°. In contrast, ETA and ESA were found to cocrystallize in an orthorhombic system with space group P212121, with the following lattice parameters and angles (ETA, ESA): a = 6.18 Å, 6.19 Å; b = 15.45 Å, 15.47 Å; and c = 21.91 Å, 21.96 Å; α = 89.98°, 90°; β = 90.03°, 90°; and γ = 90°, 90°. The structure formed with the organic acids were consistent with the prior study [15].

Alterations in the PXRD pattern of the solid product obtained following cocrystallization, compared to the starting materials, confirm the formation of a new solid phase [28]. As shown in Figure 2, the cocrystals showed PXRD patterns distinct from those of the API, coformers, and other cocrystals. The API exhibited characteristic reflections at 15.72°, 23.40°, and 32.96°. The cocrystals showed shifted peaks compared to their parent and guest crystals, suggesting significant changes in their internal structures and crystal morphology [29]. The ezetimibe/succinic acid cocrystals formed at 1:1 and 1:2 ratios showed exceptionally low yields, with distinct PXRD patterns compared to other cocrystals and significantly reduced peak intensities. EBA cocrystals (1:2) revealed unique peaks at 12.4°, 16.7°, and 24.9°, confirming new crystalline phases absent in API and coformers. For Cocrystal I (ezetimibe/benzoic acid), the monoclinic P2_1_/n symmetry (a = 5.42 Å, b = 5.05 Å, c = 21.61 Å) aligns with experimental peaks at critical angles (2θ = 12.4°, 16.7°). Similar approaches were applied to Cocrystal II (orthorhombic) and Cocrystal III (orthorhombic). Crystallographic parameters and multi-technique analyses indicate layered hydrogen-bonded networks, with lattice geometries dictating dissolution and stability behaviors. For Cocrystal I, alternating hydrophobic/hydrophilic layers likely mediate bile acid interactions during dissolution, as evidenced by in vitro sink conditions (Section 3.6). The prior studies suggest that -OH group of ezetimibe and O group of organic acids have high probability of supramolecular hetero-synthon formation (Figure 3) [30].

**Table 3:** Crystallographic properties (space group, crystal structure, and lattice parameters) of Cocrystal I (ezetimibe/benzoic acid), Cocrystal II (ezetimibe/tartaric acid), and Cocrystal III (ezetimibe/succinic acid).

Property	Cocrystal I (Benzoic Acid)	Cocrystal II (Tartaric Acid)	Cocrystal III (Succinic Acid)
Mol. Wt.	Ezetimibe: 404.45 g/mol	Ezetimibe: 404.45 g/mol	Ezetimibe: 404.45 g/mol
	Benzoic acid: 122.12 g/mol	Tartaric acid: 150.09 g/mol	Succinic acid: 118.09 g/mol
Space group	P2_1_/n	P212121	P212121
	Monoclinic	Orthorhombic	Orthorhombic
	a = 5.42 Å	a = 6.18 Å	a = 6.19 Å
	b = 5.05 Å	b = 15.45 Å	b = 15.47 Å
	c = 21.61 Å	c = 21.91 Å	c = 21.96 Å
	α = 90.00°	α = 89.98°	α = 90.00°
	β = 95.95°	β = 90.03°	β = 90.00°
	γ = 90.00°	γ = 90.00°	γ = 90.00°
Z (Units/cell)	4	4	4

### 3.4. Stoichiometry and Stability in Cocrystal Systems

The stoichiometric relationship between an API and its coformer is a critical determinant of cocrystal stability. A 1:1 API–coformer ratio typically maximizes stability via balanced hydrogen bonding and lattice packing, whereas deviations often cause biphasic systems or hydrate formation. When API exceeds the 1:1 molar ratio, a two-phase system emerges where cocrystal coexists with unreacted API due to coformer limitations. Excess API may precipitate (detectable via PXRD), compromising dissolution uniformity through altered solubility. Conversely, surplus coformer destabilizes the cocrystal lattice, promoting solvates/hydrates as extra molecules compete for hydrogen-bonding sites or introduce solvents. In hygroscopic environments, this facilitates hydrate formation through hydrophilic domains, altering mechanical properties and accelerating degradation under stress. Deviations from 1:1 stoichiometry necessitate stringent process controls during crystallization to avoid phase impurities. Excess phases may act as nucleation sites for recrystallization, requiring stability studies under ICH guidelines. Coformer solubility in specific solvents can mitigate or exacerbate stoichiometric imbalances, influencing phase outcomes. By aligning stoichiometry with thermodynamic stability, researchers can design cocrystals with predictable behavior, ensuring consistent performance in final dosage forms.

**Figure 4:**
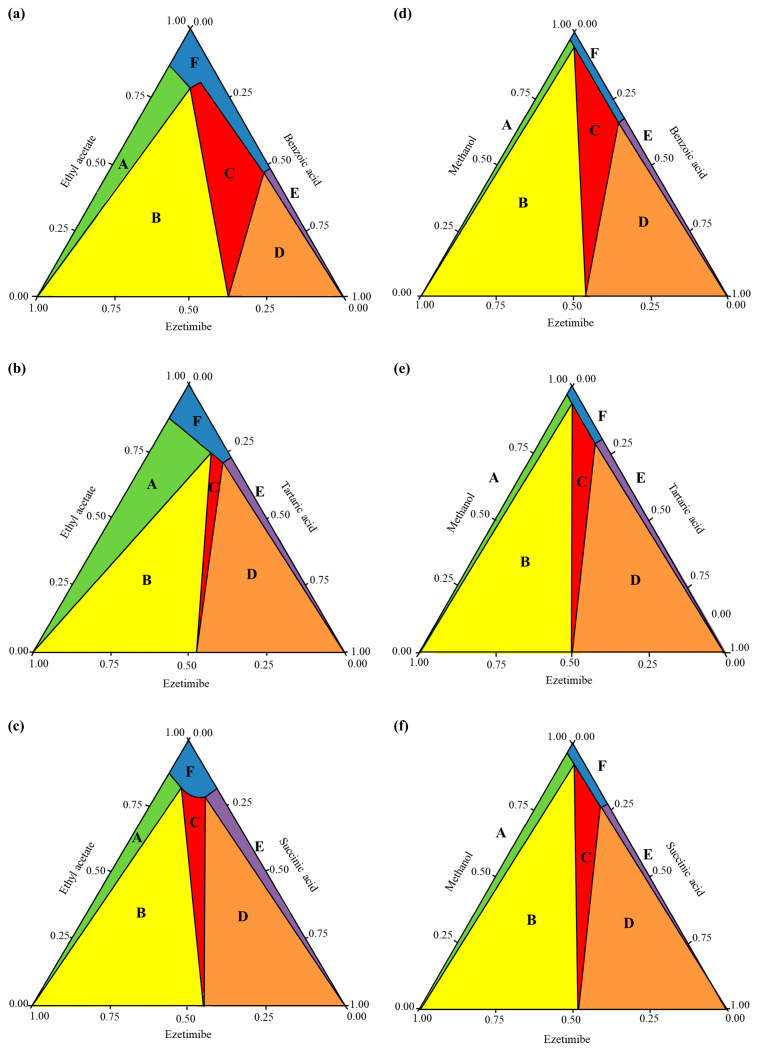
Cocrystal phase diagrams of (**a**) ezetimibe/benzoic acid, (**b**) ezetimibe/tartaric acid, and (**c**) ezetimibe/succinic acid cocrystals prepared using solvent evaporation method; and (**d**) ezetimibe/benzoic acid, (**e**) ezetimibe/tartaric acid, and (**f**) ezetimibe/succinic acid cocrystals prepared using anti-solvent precipitation method.

This study established phase diagrams by validating experimental melting temperatures ($T_{m}$, measured via DSC) vs. theoretical predictions derived from the simplified Schröder-van Laar equation. For EBA and ESA systems, the predicted $T_{m}$ values were consistently lower than experimental $T_{m}$ values across all ratios, confirming the successful formation of cocrystals through both SEV and SAS methods. However, small deviations were observed in ETA systems, specifically SEV (1:0.5, 1:1 ratios) and SAS (1:0.5), necessitating stringent process controls during preparation. Confirmation of cocrystals was obtained through SCXRD, which identified monoclinic and orthorhombic lattice structures characteristic of stable cocrystals. Phase diagrams typically represent equilateral triangles, where compositions are represented by points. These compositions are determined by projecting the points onto the sides of the triangle [31]. The COSMO quick program (Biovia, San Diego, CA, USA) was used to construct the phase diagrams of the cocrystals (Figure 4), with Figure 4a–c specifically depicting the cocrystal formation of ezetimibe with benzoic acid, tartaric acid, or succinic acid, using the SEV method in ethyl acetate at 70 °C. Similarly, Figure 4d–f shows the phase diagrams of the cocrystals formed using SAS method (MeOH, ezetimibe, and coformers). The phase diagrams showed a broad spectrum of cocrystal formation (highlighted in red and labeled ‘C’) with benzoic acid, which was followed by tartaric acid and then succinic acid in terms of formation tendency. API–coformer at 1:1 ratio maximized stability via balanced hydrogen bonding; deviations caused phase impurities. The formation of EBA cocrystals is considered highly likely with the proposed solvent system, molar ratios, and coformers. The formation of ETA and ESA cocrystals is relatively less probable and more challenging. However, under well-controlled conditions, these cocrystals can still be obtained, as demonstrated by the crystallographic analysis presented later.

### 3.5. Solubility

**Figure 5:**
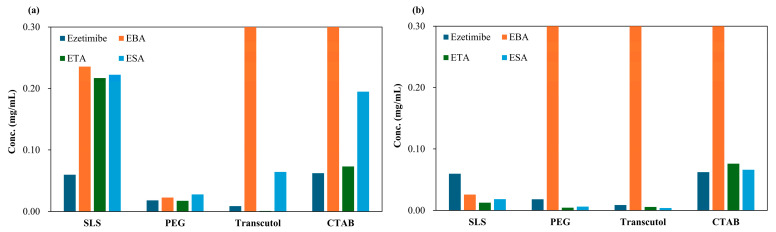
Solubility of ezetimibe/benzoic acid, ezetimibe/tartaric acid, and ezetimibe/succinic acid cocrystals, along with ezetimibe compound, evaluated in 0.5% *w*/*v* SLS, 0.5% *w*/*v* PEG, 0.5% *w*/*v* Transcutol^®^, and 0.1% *w*/*v* CTAB. The samples were prepared using (**a**) solvent evaporation and (**b**) anti-solvent precipitation methods.

To quantitatively assess solubility and in vitro release profiles, an ezetimibe calibration in a range of 1.95 μg/mL to 31.25 μg/mL showed a linear curve (Figure S2). The curve showed a correlation coefficient (R^2^) of 0.9994, with a limit of quantification (LOQ) of 0.04 µg/mL and a limit of detection (LOD) of 0.01 µg/mL (Table S4). When a hydrophilic coformer is introduced into the cocrystals of hydrophobic API, both the solubility and dissolution are enhanced compared to the parent compound. The solubility of the cocrystals is shown in Figure 5. EBA cocrystals displayed the greatest solubility enhancement, with up to 64-fold increase (SEV method), while tartaric and succinic acid cocrystals provided modest gains (3–13 fold). In general, the benzoic acid-based cocrystals displayed high solubility. The increase in solubility could be due to decreased relative free energy of solvation [32].

**Figure 6:**
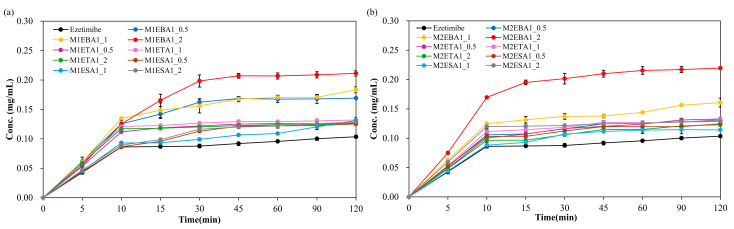
In vitro release of ezetimibe/benzoic acid, ezetimibe/tartaric acid, and ezetimibe/succinic acid cocrystals, along with ezetimibe compound, in 0.45% *w*/*v* SLS 0.05 M phosphate buffer (pH 4.5). The samples were prepared using (**a**) solvent evaporation and (**b**) anti-solvent precipitation methods.

The enhanced solubility of cocrystals has been associated with improvements in dissolution and bioavailability. EBA cocrystals (SEV) showed 64-fold solubility gain, outperforming ETA (3–13×) and ESA (3×) which is consistent with the dissolution (in vitro release profile), ranked in order of EBA > ETA > ESA. The prepared cocrystals of ezetimibe indeed displayed higher solubility than the parent compound. To assess whether this increased solubility enhances the dissolution, in vitro release was assessed using biorelevant media containing 0.45% *w*/*v* SLS in 0.05 M acetate buffer (pH 4.5) (Figure 6). M1EBA, M2EBA, and M1ETA showed significantly improved release profiles (*p*-value < 0.05). Among the cocrystals, EBA1_2 prepared using the SEV and SAS methods displayed the highest dissolution rate. Notably, M2EBA1_2 showed a dissolution rate of 0.22 mg/mL, which is approximately twice that of ezetimibe (89% vs. 45% at 120 min). The dissolution rates of ETA and ESA were comparable, and both showed improved in vitro release profiles compared to the API itself (*p*-value < 0.05). This was consistent with the solubility results and attributed to the lower melting points of the cocrystals: benzoic acid (123 °C) < ezetimibe (162 °C) < tartaric acid (172 °C) < succinic acid (188 °C). The enhanced dissolution rate of ezetimibe from its cocrystals was attributed to alterations in its crystallinity, particle size, morphology, and crystalline properties, all of which contributed to increased solubility in the dissolution medium [28,33].

### 3.6. Morphology

**Figure 7:**
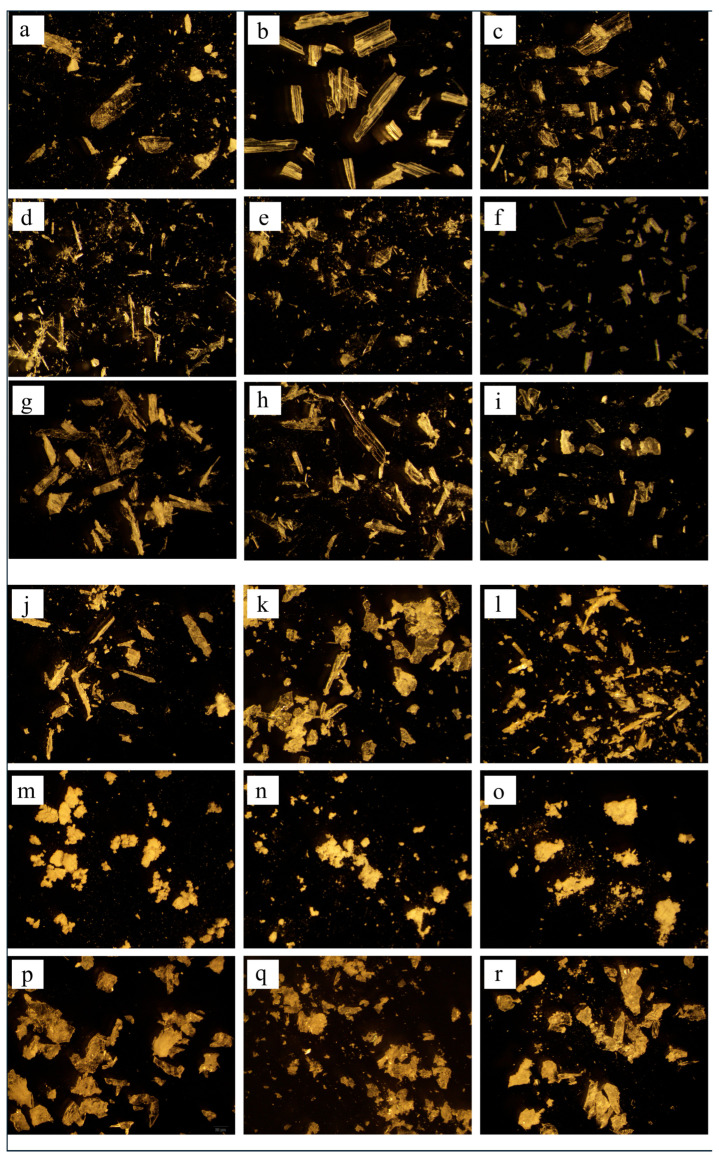
Microscopic images of ezetimibe/benzoic acid cocrystals (at 20× magnification) prepared using (**a**–**c**) solvent evaporation and (**j**–**l**) anti-solvent precipitation methods at 1:0.5, 1:1, 1:2 ratios. Images of the ezetimibe/tartaric acid cocrystals using the (**d**–**f**) solvent evaporation and (**m**–**o**) anti-solvent precipitation methods at 1:0.5, 1:1, 1:2 ratios. Images of the ezetimibe/succinic acid cocrystals using the (**g**–**i**) solvent evaporation and (**p**–**r**) anti-solvent precipitation methods at 1:0.5, 1:1, and 1:2 ratios.

**Figure 8:**
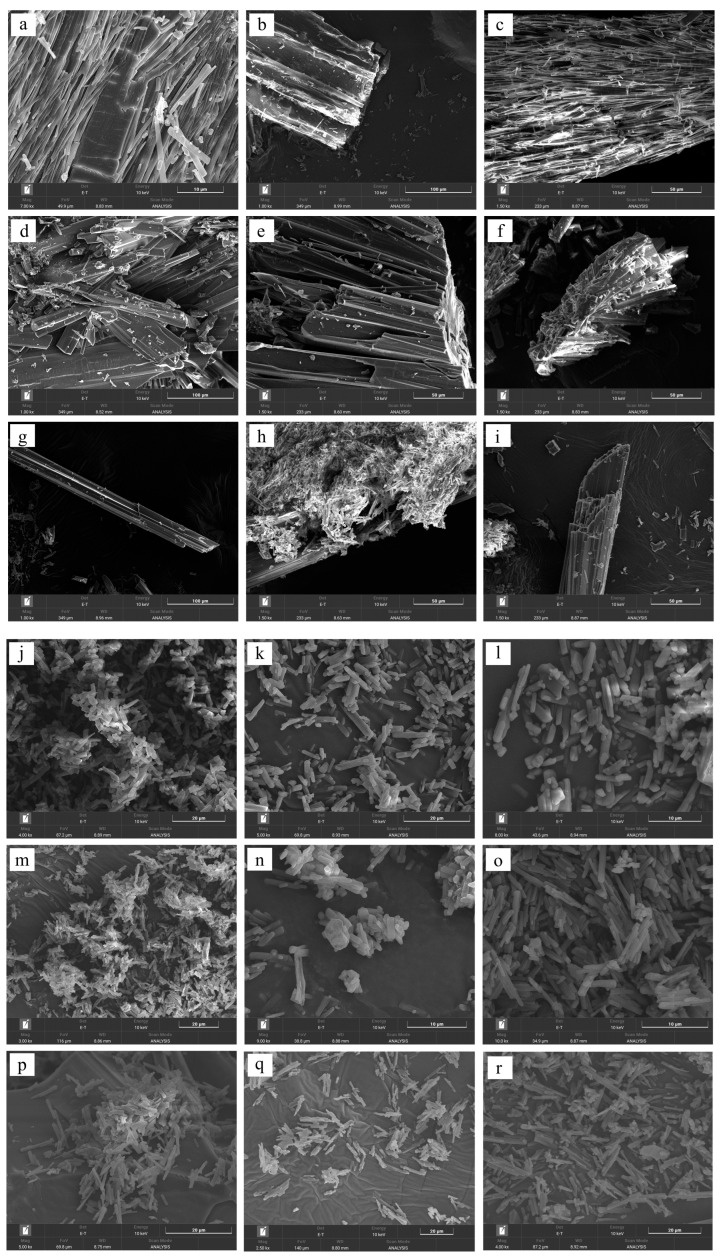
SEM images of ezetimibe/benzoic acid cocrystals prepared using the (**a**–**c**) solvent evaporation and (**j**–**l**) anti-solvent precipitation methods at 1:0.5, 1:1, 1:2 ratios. Images of the ezetimibe/tartaric acid cocrystals using the (**d**–**f**) solvent evaporation and (**m**–**o**) anti-solvent precipitation methods at 1:0.5, 1:1, 1:2 ratios. Images of the ezetimibe/succinic acid cocrystals using the (**g**–**i**) solvent evaporation and (**p**–**r**) anti-solvent precipitation methods at 1:0.5, 1:1, 1:2 ratios.

The surface characteristics of the cocrystals were investigated by conventional microscopic imaging (Figure 7) and SEM (Figure 8). SEV produced larger crystals (50–100 μm), while SAS yielded smaller, irregular particles (5–20 μm) due to rapid supersaturation (Figure 7 and Figure 8). This enables stable crystal growth as the solute concentration increases and typically leads to a larger crystal size and improved crystallinity. On the other hand, the SAS method involves the use of an additional solvent, resulting in rapid supersaturation and immediate crystallization of the solute. This process limits the time needed for sufficient crystal formation, leading to the production of smaller crystals.

### 3.7. Effects of SEV and SAS Methods on Cocrystals

Previously, an average mean diameter of approximately 4.66 µm was reported for particles produced using the SAS method. Particles produced using the traditional SEV method have been reported to have an average mean diameter of approximately 38.09 µm [34]. As aforementioned, a slower evaporation rate tends to yield larger and more uniform crystals, enhancing their stability and reproducibility in applications [35]. The SEV method offers more effective control over the crystallization process compared to the SAS method, where the introduction of an anti-solvent rapidly increases supersaturation, resulting in prompt nucleation of crystalline particles [36]. The two methods produce same cocrystals and only exhibit fundamental differences in particle formation [37].

### 3.8. Impact of the Drying Rate on Cocrystals

For APIs with absorption limited by dissolution rate, employing a cocrystal strategy can enhance their bioavailability by improving their dissolution rates [38]. The presence of evaporating droplets can impact cocrystal formation by altering the drying rate during crystallization. When drying occurs over a sufficient period, crystal growth proceeds more regularly and stably, enhancing the integrity of the crystal structure. In contrast, rapid drying can result in incomplete crystal formation, leading to irregular shapes with weaker bonding.

The drying rate is dependent on temperature and humidity [39]. Controlling this factor is common and effective for increasing both solubility (*C_s_*) and dissolution rates (*dC*/*dt*) of both acidic and basic APIs. This factor also impacts the wettability, which in turn influences the diffusion layer thickness (*h*) [40]. This phenomenon can enhance solubility by several orders of magnitude, significantly enhancing the dissolution rate [41].

### 3.9. In Vivo Pharmacokinetic Evaluation

The pharmacokinetic evaluation of ezetimibe cocrystals showed dose-dependent absorption, probably influenced by formulation design and physiological conditions. CRYS101, CRYS102, CRYS103, and CRYS104 are ezetimibe, ezetimibe/benzoic acid (1:2 ratio), M1EBA1_2 (SEV), and M2EBA1_2 (SAS), respectively. At the optimized 30 mg/kg dose, EBA cocrystals (SEV, 1:2 ratio) showed 3× higher Cmax and 4× AUC vs. pure ezetimibe (Table 4, *p*-value < 0.05). Our Cmax surpasses Wang et al.’s (2024) 1.8-fold enhancement with curcumin cocrystals, likely due to optimized hydrogen bonding in P21/n lattices [24].The delayed time to peak concentration (T_max_ = 2.83 ± 2.75 h vs. 1.33 ± 0.76 h) correlated with in vitro dissolution data showing sustained release (89% at 120 min vs. 45% ezetimibe), suggesting cocrystal lattice stability prolongs gastric retention. While dissolution showed solubility enhancement, the 43.75% of samples were below the quantification limit (10 mg/kg) and exhibited sub-optimal bioavailability. Probably it may have prevented micellar solubilization due to bile acid sequestration effect. Another probable reason could be the rapid dissolution which overwhelms absorption capacity and trigger API recrystallization. This aligns with a prior study’s observations of dose-dependent absorption threshold [42,43].

The monoclinic crystal lattice (space group P2_1_/n) provided thermodynamic stability but triggered size-dependent dissolution challenges. SAS batches showed faster initial release (45% at 15 min) vs. SEV (22%), yet in vivo data revealed paradoxical exposure patterns where some 30 mg/kg subjects underperformed 10 mg/kg counterparts. The cocrystal production with SAS method, targeting size range of 50–100 μm could balance dissolution rate and prevent supersaturation precipitation. The overall findings convincingly position BA cocrystallization as a viable strategy for BCS class II drugs but emphasize the need for integrated particle design, dose optimization, and biorelevant dissolution modeling to translate in vitro advantages into consistent in vivo performance. While the method validation met regulatory criteria (R^2^ = 0.995, accuracy 88–103%), the frequent BQL occurrences in vivo indicate that 1 ng/mL LLOQ may be inadequate for preclinical studies, necessitating either dose increment or analytical sensitivity improvements.

## 4. Conclusions

This study demonstrates, for the first time, that benzoic acid–ezetimibe cocrystals (1:2, SEV) provide superior solubility (64×), dissolution (2×), and bioavailability (3–4×). These findings highlight cocrystallization as a rational formulation approach to overcome solubility barriers in BCS Class II drugs, with clinical translation potential. The DSC thermograms, FTIR spectra, PXRD pattern, and single crystal analysis confirmed a distinct peak, hydrogen bonding, and crystalline lattice, confirming the formation of cocrystals. The cocrystals represent a promising strategy to enhance the solubility of a poorly soluble drug.

## Figures and Tables

**Table 1:** DSC thermogram analysis of ezetimibe, coformers, cocrystals (ezetimibe/benzoic acid, ezetimibe/tartaric acid, and ezetimibe/succinic acid), and their physical mixtures.

			1st T_m_		2nd T_m_	
		°C	ΔT_m_ (°C)	J/g	°C	J/g
API	Ezetimibe	162.36	-	87.04	-	-
Coformer	Benzoic acid	123.19	39.17	148.9	-	-
SEV method	M1EBA1_0.5	110.24	52.12	50.05	-	-
	M1EBA1_1	111.68	50.68	120.5	-	-
	M1EBA1_2	111.06	51.3	107.8	-	-
SAS method	M21EBA1_0.5	109.76	52.6	26.89	119.27	70.56
	M2EBA1_1	109.65	52.71	18.04	120.44	96.95
	M2EBA1_2	109.22	53.14	10.18	120.76	109.5
Physical mixture	PMEBA1_0.5	110.24	52.12	8.277	162.44	68.03
	PMEBA1_1	111.14	51.22	33.87	122.38	56.96
	PMEBA1_2	110.43	51.93	56.01	119.55	22.84
Coformer	Tartaric acid	172.67	−10.31	251.2	-	-
SEV method	M1ETA1_0.5	160.24	2.12	82.41	167.25	13.83
	M1ETA1_1	159.32	3.04	68.19	166.70	35.06
	M1ETA1_2	156.38	5.98	13.31	171.18	182.4
SAS method	M21ETA1_0.5	154.14	8.22	61.87	162.58	33.18
	M2ETA1_1	154.22	8.14	39.60	165.35	89.38
	M2ETA1_2	154.67	7.69	29.09	168.12	144.3
Physical mixture	PMETA1_0.5	158.11	4.25	11.478	170.59	143.8
	PMETA1_1	157.01	5.35	12.39	171.33	183.4
	PMETA1_2	156.84	5.52	12.09	171.01	188.9
Coformer	Succinic acid	188.91	−26.55	299.6	-	-
SEV method	M1ESA1_0.5	151.82	10.54	17.62	183.22	111.1
	M1ESA1_1	155.57	6.79	24.83	185.36	154.0
	M1ESA1_2	150.92	11.44	2.79	187.38	231.8
SAS method	M21ESA1_0.5	150.39	11.97	4.55	186.44	174.8
	M2ESA1_1	-	-	-	188.08	245.4
	M2ESA1_2	149.55	12.81	1.78	187.06	223.3
Physical mixture	PMESA1_0.5	152.03	10.33	21.01	182.97	118.8
	PMESA1_1	152.25	10.11	52.72	183.65	70.14
	PMESA1_2	151.16	11.2	22.04	185.89	177.1

API—Active pharmaceutical ingredient, SEV—Solvent evaporation method, SAS—Solvent anti-solvent method.

**Table 2:** Significant functional groups observed in the FTIR spectra of ezetimibe, Cocrystal I (ezetimibe/benzoic acid), Cocrystal II (ezetimibe/tartaric acid), and Cocrystal III (ezetimibe/succinic acid).

	Cocrystal I (Benzoic Acid)		Cocrystal II (Tartaric Acid)		Cocrystal III (Succinic Acid)		Ezetimibe	Functional Group
	SEV	SAS	SEV	SAS	SEV	SAS		
1:0.5	3224.89	3228.57	-	-	3230.96	3228.57	3264.09	O-H
	2912.29	2912.94	-	-	2912.90	2912.94	2928.09	C-H
	-	1714.46	1713.08	1715.16	1714.11	1714.46	1726.06	C=O
	1507.88	1507.70	1507.63	1507.92	1507.89	1507.70	1507.08	C=C
	1218.85	1218.67	1218.64	1219.11	1200.53	1218.67	1212.71	C-F
1:1	-	3234.65	-	3406.25	3220.01	-	3264.09	O-H
	-	2912.69	-	-	2912.97	2930.36	2928.09	C-H
	1713.53	1713.42	1713.46	1715.19	1713.14	-	1726.06	C=O
	1507.94	1507.58	1507.53	1507.99	1507.81	1506.62	1507.08	C=C
	1219.04	1218.49	1217.82	1218.20	1200.78	1196.61	1212.71	C-F
1:2	-	-	3324.29	3396.99	3230.96	-	3264.09	O-H
	-	2828.22	-	-	2928.02	2912.70	2928.09	C-H
	1712.60	1678.48	1714.94	1713.99	1724.75	1713.97	1726.06	C=O
	1507.76	1508.94	1507.93	1508.27	1506.87	1507.74	1507.08	C=C
	1219.82	1220.23	1217.57	1217.08	1212.61	1197.52	1212.71	C-F

SEV—Solvent evaporation method, SAS—Solvent anti-solvent method.

**Table 4:** Summary of mean (±SD) in vivo pharmacokinetic parameters for four ezetimibe formulations (CRYS101–CRYS104) in Sprague Dawley rats.

Formulation	Dose (mg/kg)	C_max_ (ng/mL)	T_max_ (h)	AUC_last_ (ng·h/mL)	AUC_inf_ (ng·h/mL)
CRYS101	10	6.73 ± 4.29	1.33 ± 0.76	11.03 ± 8.63	41.83 ^#^
CRYS102	10	5.50 ± 4.23	1.13 ± 0.48	15.26 ± 7.08	31.08 ^#^
CRYS103	10	18.38 ± 9.52	2.83 ± 2.75	40.36 ± 30.94	17.75 ^#^
CRYS104	10	4.30 ± 3.25	1.00 ^#^	9.62 ± 8.82	ND

ND: Not determined, below quantification limit (1 ng/mL). ^#^: SD not determined.

## Data Availability

Data are contained within the article and Supplementary Materials.

## Supplementary Materials

The following supporting information can be downloaded at: https://www.mdpi.com/article/10.3390/pharmaceutics17111399/s1, Table S1. System Suitability testing conducted with four parameters (retention time, peak area, tailing factor, and theoretical plates) for ezetimibe analysis method along with acceptance criteria; Table S2. Accuracy and precision parameters for the analytical method validation of ezetimibe assay determination; Table S3. Recovery parameter for the analytical method validation of ezetimibe assay determination; Table S4. The parameters of the calibration curve correspond to the linearity range for ezetimibe; Figure S1. Preparation of ezetimibe cocrystals using solvent evaporation and anti-solvent precipitation methods; Figure S2. Ezetimibe calibration curves over the range of 1.95 µg/mL to 31.25 µg/mL; Figure S3. DSC thermograms of (A) ezetimibe/benzoic acid, (B) ezetimibe/tartaric acid, and (C) ezetimibe/succinic acid cocrystals. The thermograms of the parent compound and their physical mixtures, obtained using solvent evaporation and anti-solvent precipitation methods at 1:0.5, 1:1, and 1:2 ratios.

## References

[1] Kavanagh O.N. (2024). An analysis of multidrug multicomponent crystals as tools for drug development. J. Control. Release.

[2] Maharjan R., Jeong J., Bhujel R., Kim M.-S., Han H.-K., Kim N.A., Jeong S.H. (2022). Correlation of Solubility Thermodynamics of Glibenclamide with Recrystallization and In Vitro Release Profile. Molecules.

[3] Bhalani D.V., Nutan B., Kumar A., Singh Chandel A.K. (2022). Bioavailability enhancement techniques for poorly aqueous soluble drugs and therapeutics. Biomedicines.

[4] Aakeröy C.B., Fasulo M.E., Desper J. (2007). Cocrystal or salt: Does it really matter?. Mol. Pharm..

[5] Ghanavati M.A., Khalili B., Alberico D., Rohani S. (2025). Machine learning-driven discovery of multicomponent pharmaceutical solid forms via DualNet: Confidence-aware prediction and ranking of salts and cocrystals. Int. J. Pharm..

[6] Haneef J., Amir M., Sheikh N.A., Chadha R. (2023). Mitigating drug stability challenges through cocrystallization. AAPS PharmSciTech.

[7] Shan N., Zaworotko M.J. (2008). The role of cocrystals in pharmaceutical science. Drug Discov. Today.

[8] D’Abbrunzo I., Gigli L., Demitri N., Sabena C., Nervi C., Chierotti M.R., Bertoni S., Škorić I., Häberli C., Keiser J. (2025). Higher-order multicomponent crystals as a strategy to decrease the IC50 parameter: The case of praziquantel, niclosamide and acetic acid. J. Drug Deliv. Sci. Technol..

[9] Duggirala N.K., Perry M.L., Almarsson Ö., Zaworotko M.J. (2016). Pharmaceutical cocrystals: Along the path to improved medicines. Chem. Commun..

[10] Kavanagh O.N., Croker D.M., Walker G.M., Zaworotko M.J. (2019). Pharmaceutical cocrystals: From serendipity to design to application. Drug Discov. Today.

[11] Share Mohammadi H., Haghighi Asl A., Khajenoori M. (2024). Preparation of Aprepitant nanoparticles using subcritical water anti-solvent technology. Powder Technol..

[12] Pu Y., Li Y., Wang D., Foster N.R., Wang J.-X., Chen J.-F. (2017). A green route to beclomethasone dipropionate nanoparticles via solvent anti-solvent precipitation by using subcritical water as the solvent. Powder Technol..

[13] Shimpi M.R., Childs S.L., Boström D., Velaga S.P. (2014). New cocrystals of ezetimibe with L-proline and imidazole. CrystEngComm.

[14] Prajapati P., Pandey J., Tandon P., Sinha K., Shimpi M.R. (2022). Molecular Structural, Hydrogen Bonding Interactions, and Chemical Reactivity Studies of Ezetimibe-L-Proline Cocrystal Using Spectroscopic and Quantum Chemical Approach. Front. Chem..

[15] Childs S.L., Chyall L.J., Dunlap J.T., Smolenskaya V.N., Stahly B.C., Stahly G.P. (2004). Crystal engineering approach to forming cocrystals of amine hydrochlorides with organic acids. Molecular complexes of fluoxetine hydrochloride with benzoic, succinic, and fumaric acids. J. Am. Chem. Soc..

[16] Grecu T., Hunter C.A., Gardiner E.J., McCabe J.F. (2014). Validation of a computational cocrystal prediction tool: Comparison of virtual and experimental cocrystal screening results. Cryst. Growth Des..

[17] Abramov Y.A., Shah H.S., Michelle C., Wan Z., Xie T., Kuang S., Wang J. (2025). Computational and Experimental Cocrystal Screening of Tiopronin and Dapagliflozin APIs: Development and Validation of a New Virtual Screening Model. Cryst. Growth Des..

[18] Ravi M., Julu T., Kim N.A., Park K.E., Jeong S.H. (2021). Solubility Determination of c-Met Inhibitor in Solvent Mixtures and Mathematical Modeling to Develop Nanosuspension Formulation. Molecules.

[19] Ronik D.F., Hosni A.P., Brancalione R.C., Biscaia I.F., Bernardi L.S., Oliveira P.R.d. (2024). Synthesis and characterization of ezetimibe pharmaceutical cocrystal: A reaction crystallization method to improve physicochemical properties and hypolipemic activity evaluation. J. Braz. Chem. Soc..

[20] Maharjan R., Lee J.C., Kim N.A., Jeong S.H. (2021). Preparation of seeded granules to improve mechanical properties and various drug loading for pharmaceutical application. Powder Technol..

[21] Vogt F.G., Roberts-Skilton K., Kennedy-Gabb S.A. (2013). A solid-state NMR study of amorphous ezetimibe dispersions in mesoporous silica. Pharm. Res..

[22] Indrayanto G., Syahrani A., Rahman A., Tanudjojo W., Susanti S., Yuwono M., Ebel S. (1999). Benzoic acid. Analytical Profiles of Drug Substances and Excipients.

[23] Shen J., Zheng J., Che Y., Xi B. (2003). Growth and properties of organic nonlinear optical crystals: L-tartaric acid–nicotinamide and d-tartaric acid–nicotinamide. J. Cryst. Growth.

[24] Wang Y., Wu Y., Wu L., Wang J., Huang C., Leng Y. (2024). Determination and molecular simulation of ternary solid–liquid phase equilibrium of succinic acid+ maleic acid+ water from 283.15 K to 333.15 K. J. Chem. Thermodyn..

[25] Gong N., Yu H., Wang Y., Xing C., Hu K., Du G., Lu Y. (2020). Crystal structures, stability, and solubility evaluation of a 2: 1 diosgenin–piperazine cocrystal. Nat. Prod. Bioprospect..

[26] Anand R., Nanda A. (2022). Formulation and Evaluation of Cocry-stals of a Bcs Class Ii Drug Using Glycine As Coformer. Int. J. Appl. Pharm..

[27] Schultheiss N., Newman A. (2009). Pharmaceutical cocrystals and their physicochemical properties. Cryst. Growth Des..

[28] Sugandha K., Kaity S., Mukherjee S., Isaac J., Ghosh A. (2014). Solubility enhancement of ezetimibe by a cocrystal engineering technique. Cryst. Growth Des..

[29] Mulye S.P., Jamadar S.A., Karekar P.S., Pore Y.V., Dhawale S.C. (2012). Improvement in physicochemical properties of ezetimibe using a crystal engineering technique. Powder Technol..

[30] Bhandari J., Kanswami N. (2020). Nano co-crystal engineering technique to enhance the solubility of Ezetimibe. J. Young Pharm..

[31] Ainouz A., Authelin J.-R., Billot P., Lieberman H. (2009). Modeling and prediction of cocrystal phase diagrams. Int. J. Pharm..

[32] Surov A.O., Ramazanova A.G., Voronin A.P., Drozd K.V., Churakov A.V., Perlovich G.L. (2023). Virtual Screening, Structural Analysis, and Formation Thermodynamics of Carbamazepine Cocrystals. Pharmaceutics.

[33] Martínez-Alejo J.M., Höpfl H., Herrera-Ruiz D., Morales-Rojas H. (2025). A Portfolio of Ciprofloxacin Hydrochloride Ionic Cocrystals with Phenolic Acids for Tailoring Dissolution and Solubility. Cryst. Growth Des..

[34] Tjandrawinata R.R., Hiendrawan S., Veriansyah B. (2019). Processing paracetamol-5-nitroisophthalic acid cocrystal using supercritical CO2 as an anti-solvent. Int. J. Appl. Pharm.

[35] Kumar R., Siril P.F., Soni P. (2014). Preparation of Nano-RDX by Evaporation Assisted Solvent Antisolvent Interaction. Prop. Explos. Pyrotech..

[36] Gadhiya D.T., Patel J.K., Bagada A.A. (2021). An impact of nanocrystals on dissolution rate of Lercanidipine: Supersaturation and crystallization by addition of solvent to antisolvent. Future J. Pharm. Sci..

[37] Yu J., Henry R.F., Zhang G.G. (2025). Cocrystal screening in minutes by solution-mediated phase transformation (SMPT): Preparation and characterization of ketoconazole cocrystals with nine aliphatic dicarboxylic acids. J. Pharm. Sci..

[38] Wang H., Zheng C., Tian F., Xiao Z., Sun Z., Lu L., Dai W., Zhang Q., Mei X. (2024). Improving the dissolution rate and bioavailability of curcumin via co-crystallization. Pharmaceuticals.

[39] Shahidzadeh N., Schut M.F., Desarnaud J., Prat M., Bonn D. (2015). Salt stains from evaporating droplets. Sci. Rep..

[40] Elder D.P., Holm R., De Diego H.L. (2013). Use of pharmaceutical salts and cocrystals to address the issue of poor solubility. Int. J. Pharm..

[41] Serajuddin A.T. (2007). Salt formation to improve drug solubility. Adv. Drug Deliv. Rev..

[42] Cheung A.K., Liu D., Peukert S., Ge H., Gai Y., Chang X. (2024). Naphthyridinone Derivatives for the Treatment of a Disease or. Disorder. Patent.

[43] Mo P., Hatanaka Y., Furukawa S., Takase M., Yamanaka S., Doi M., Kämäräinen T., Uchiyama H., Kadota K., Tozuka Y. (2024). Cocrystal formulation design of 4-Aminosalicylic acid and isoniazid via spray-drying based on a ternary phase diagram toward simultaneous pulmonary delivery. Powder Technol..

