# Supplementary material for: Cocrystallization of Ezetimibe with Organic Acids: Stoichiometric Optimization for Improved Solubility and Bioavailability
Source: Pharmaceutics. 2025 Oct 29;17(11):1399. doi: 10.3390/pharmaceutics17111399 (PMC12655579; doi:10.3390/pharmaceutics17111399)
Supplement: Supplementary file 1 [file pharmaceutics-17-01399-s001.zip › pharmaceutics-3876207-supplementary.pdf]

Supplementary Materials

Cocrystallization of ezetimibe with organic acids at different stoichiometry to improve physicochemical properties and bioavailability

Ravi Maharjan[1†], Ha Eun Park[2†], Ki Hyun Kim[3], Mansingh Chaudhary[4], Ki-Taek Kim[3,4], Minji Kim[5], Hea-Young Cho[5], Seong Hoon Jeong[1]*

[1]College of Pharmacy & Yonsei Institute of Pharmaceutical Sciences, Yonsei University, Incheon 21983, Republic of Korea

[2]College of Pharmacy, Dongguk University, Gyeonggi 10326, Republic of Korea

[3]College of Pharmacy and Natural Medicine Research Institute, Mokpo National University, Jeonnam 58554, Republic of Korea

[4]Department of Biomedicine, Health & Life Convergence Sciences, BK21 Four, Biomedical and Healthcare Research Institute, Mokpo National University, Jeonnam 58554, Republic of Korea

[5]College of Pharmacy, CHA University, Seongnam 13488, Republic of Korea

Table S1. System Suitability testing conducted with four parameters (retention time, peak area, tailing factor, and theoretical plates) for ezetimibe analysis method along with acceptance criteria.

Parameter	Acceptance Criteria	Ezetimibe	IS
Retention time (min)	RSD ≤2%	1.8 (0.8%)	2.1 (1.1%)
Peak area RSD (n = 6)	≤5%	3.2%	2.8%
Tailing factor	≤2.0	1.1	0.9
Theoretical plates	≥2000	4589	5123

Table S2. Accuracy and precision parameters for the analytical method validation of ezetimibe assay determination.

QC Level	Conc. (ng/mL)	Intra-day (*n* = 5)	Inter-day (*n* = 3 days)
		Accuracy (%)	Precision (% CV)
LLOQ	1.0	103.1 ± 3.5	9.1
Low	3.0	98.1 ± 3.5	10.2
Medium	40.0	88.5 ± 1.2	2.7
High	80.0	90.8 ± 1.8	5.9

Table S3. Recovery parameter for the analytical method validation of ezetimibe assay determination.

Component	Absolute Recovery (%)	CV (%)
Ezetimibe	85.2 ± 4.1	4.8
IS	92.3 ± 3.6	3.9

Table S4. The parameters of the calibration curve correspond to the linearity range for ezetimibe.

Parameters	Mean ± SD
Slope	44.58 ± 0.02
Intercept	0.01
Correlation coefficient (R^2)	0.9994
Limit of quantification (LOQ)	0.04 μg/mL
Limit of detection (LOD)	0.01 μg/mL

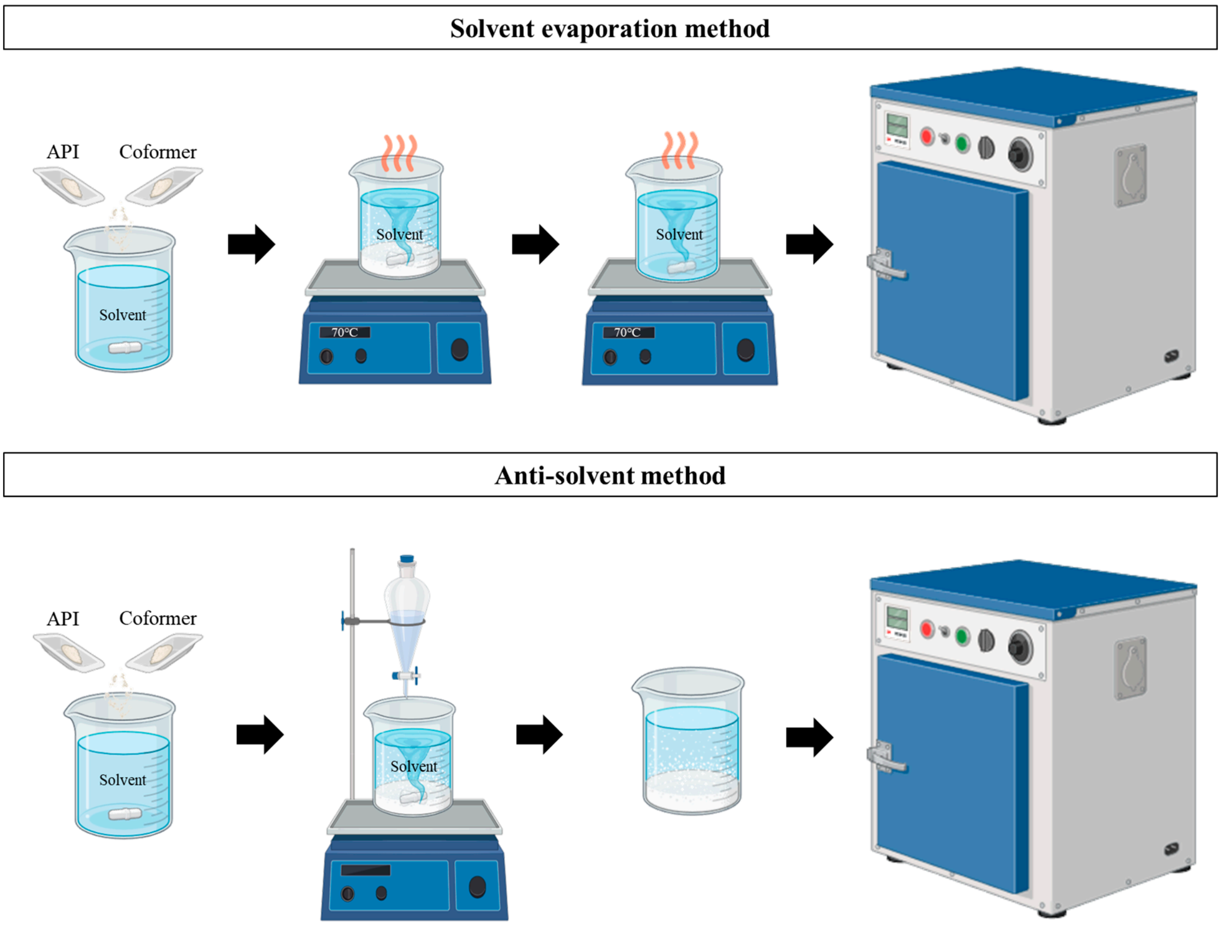

Figure S1. Preparation of ezetimibe cocrystals using solvent evaporation and anti-solvent precipitation methods.

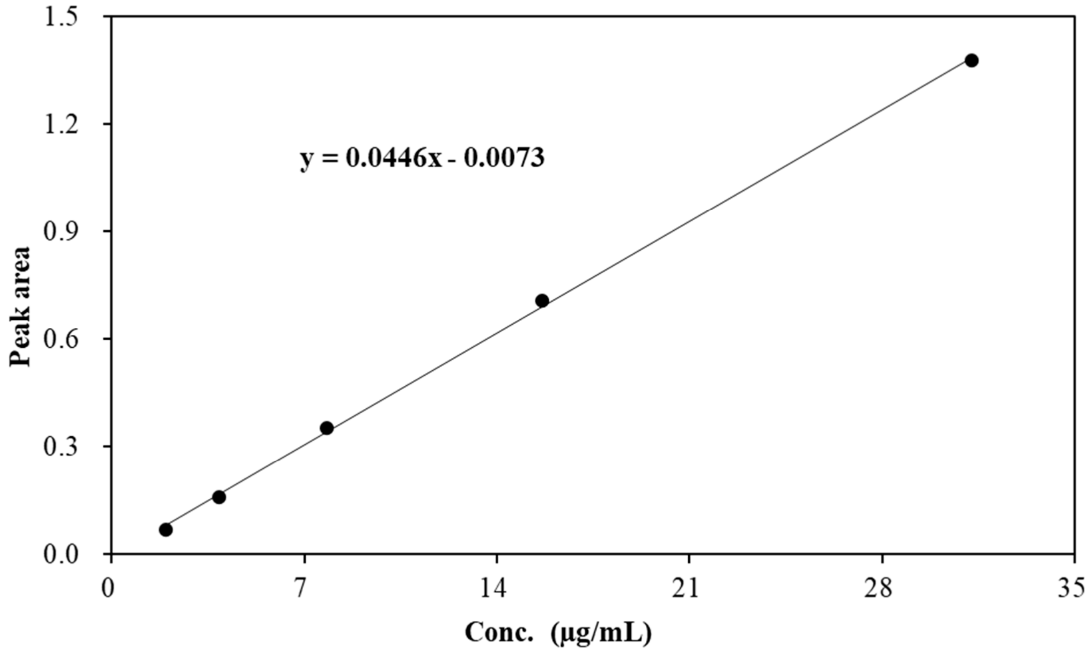

Figure S2. Ezetimibe calibration curves over the range of 1.95 µg/mL to 31.25 µg/mL.

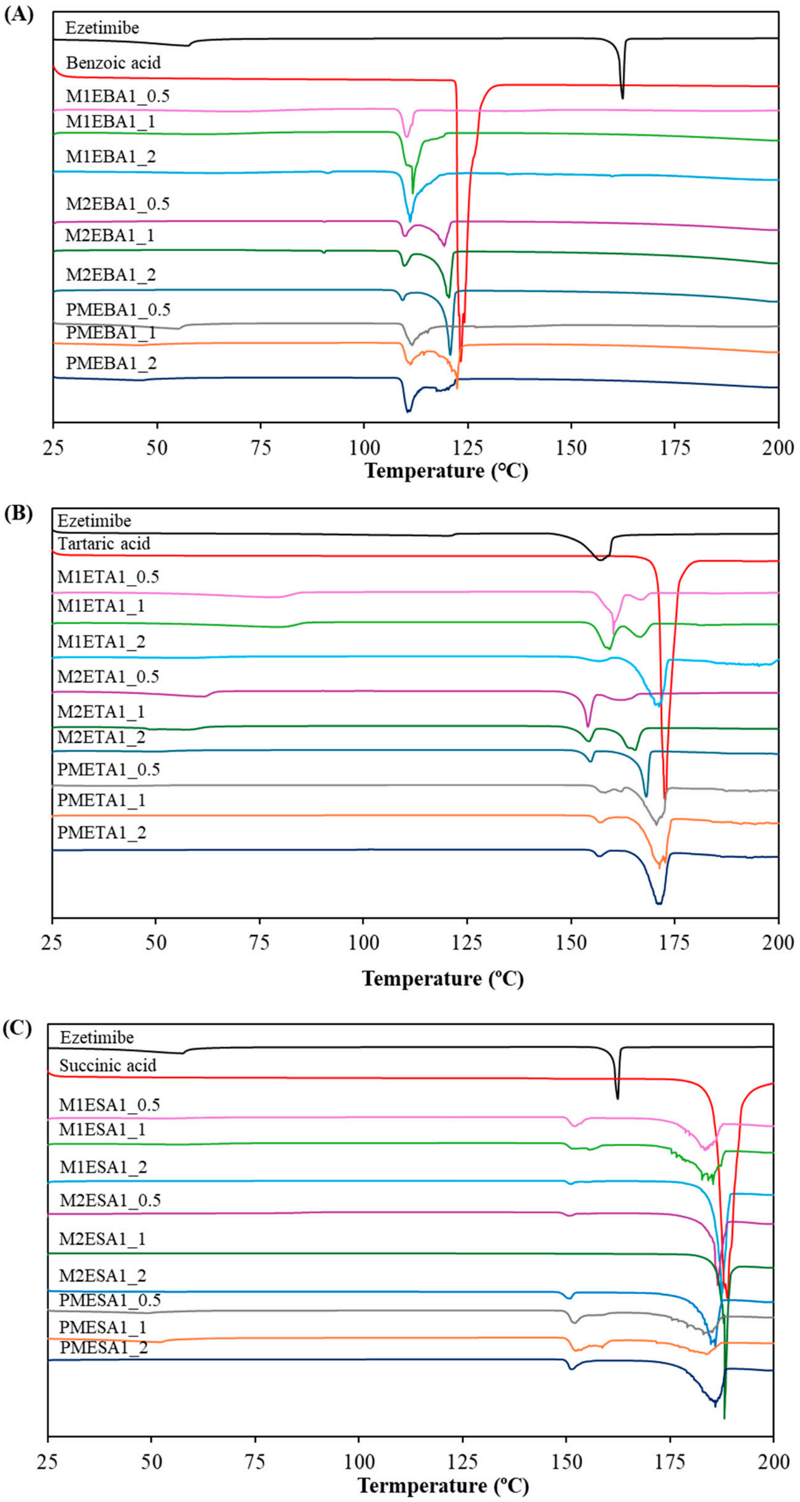

Figure S3. DSC thermograms of (A) ezetimibe/benzoic acid, (B) ezetimibe/tartaric acid, and (C) ezetimibe/succinic acid cocrystals. The thermograms of the parent compound and their physical

mixtures, obtained using solvent evaporation and anti-solvent precipitation methods at 1:0.5, 1:1, and 1:2 ratios. The ezetimibe/benzoic acid cocrystals were denoted as follows: M1EBA1_0.5, M1EBA1_1, M1EBA1_2, M2EBA1_0.5, M2EBA1_1, and M2EBA1_2. The ezetimibe/tartaric acid cocrystals were denoted as follows: M1ETA1_0.5, M1ETA1_1, M1ETA1_2, M2ETA1_0.5, M2ETA1_1, and M2ETA1_2. The ezetimibe/succinic acid cocrystals were denoted as follows: M1ESA1_0.5, M1ESA1_1, M1ESA1_2, M2ESA1_0.5, M2ESA1_1, and M2ESA1_2. The physical mixtures of ezetimibe with each coformer were denoted as follows: PMEBA1_0.5, PMEBA1_2, PMETA1_0.5, PMETA1_1, PMETA1_2, PMESA1_0.5, PMESA1_1, and PMESA1_2.

1. Bioanalytical Method Development

The calibration curve was constructed with five standards ranging from 500 ppm to 1.95 ppm. The absorbance of the standards at 233 nm was measured using a UV-VIS spectrophotometer (Optizen pop, Mecasys®, Seoul, South Korea). The stock standard solution was prepared by dissolving ezetimibe at 0.5 mg/mL in 40% MeOH. The bioanalytical method was developed using an Agilent 1290 Infinity II BioLC system coupled with an Agilent 6495D Triple-Quadrupole mass spectrometer, employing electrospray ionization (ESI). Chromatographic separation was achieved on a Kintex C18 column (2.6 µm, 100×3 mm) maintained at 30 °C, using an isocratic mobile phase of water-acetonitrile (20:80 v/v) at 0.25 mL/min flow rate. The total running time was optimized to 5 min with a 2 µL injection volume. Mass spectrometric detection utilized multiple reaction monitoring (MRM) in negative polarity mode for ezetimibe ($[M-H]^-$ m/z 408.1→271.1) and positive mode for the internal standard itraconazole ([M+H]+ m/z 705.2→391.6). Source parameters were optimized: gas temperature 290 °C with 13 L/min flow, nebulizer pressure 35 psi, and sheath gas heated to 250 °C at 11 L/min. Collision energies were set at 14 eV for ezetimibe and 48 eV for IS, achieving optimal fragmentation while maintaining signal intensity.

Sample preparation involved protein precipitation with acetonitrile (75 µL added to 20 µL plasma), followed by vortex mixing and centrifugation at 13,000 ×g for 10 min. The method demonstrated linearity ($R^2 = 0.995$) across 1-100 ng/mL, with calibration standards prepared by spiking blank plasma. Quality control samples at four concentrations (1, 3, 40, and 80 ng/mL) showed acceptable accuracy (88.47-103.08%) and precision (CV≤9.8%). The lower limit of quantification (LLOQ) was established at 1 ng/mL with 103.08% accuracy and 8.87% precision.
